# In Vivo Measurement of Hippocampal GABA_A_/cBZR Density with [^18^F]-Flumazenil PET for the Study of Disease Progression in an Animal Model of Temporal Lobe Epilepsy

**DOI:** 10.1371/journal.pone.0086722

**Published:** 2014-01-21

**Authors:** Lucy Vivash, Marie-Claude Gregoire, Viviane Bouilleret, Alexis Berard, Catriona Wimberley, David Binns, Peter Roselt, Andrew Katsifis, Damian E. Myers, Rodney J. Hicks, Terence J. O'Brien, Stefanie Dedeurwaerdere

**Affiliations:** 1 Department of Medicine, Royal Melbourne Hospital, University of Melbourne, Melbourne, Victoria, Australia; 2 Department of LifeSciences, Australian Nuclear Science and Technology Organisation, Sydney, New South Wales, Australia; 3 The Centre for Molecular Imaging, Peter MacCallum Cancer Centre, Melbourne, Victoria, Australia; 4 Department of Translational Neurosciences, University of Antwerp, Wilrijk, Belgium; University of Pécs Medical School, Hungary

## Abstract

**Purpose:**

Imbalance of inhibitory GABAergic neurotransmission has been proposed to play a role in the pathogenesis of temporal lobe epilepsy (TLE). This study aimed to investigate whether [^18^F]-flumazenil ([^18^F]-FMZ) PET could be used to non-invasively characterise GABA_A_/central benzodiazepine receptor (GABA_A_/cBZR) density and affinity *in vivo* in the post-kainic acid *status epilepticus* (SE) model of TLE.

**Methods:**

Dynamic [^18^F]-FMZ -PET scans using a multi-injection protocol were acquired in four male wistar rats for validation of the partial saturation model (PSM). SE was induced in eight male Wistar rats (10 weeks of age) by i.p. injection of kainic acid (7.5–25 mg/kg), while control rats (n = 7) received saline injections. Five weeks post-SE, an anatomic MRI scan was acquired and the following week an [^18^F]-FMZ PET scan (3.6–4.6 nmol). The PET data was co-registered to the MRI and regions of interest drawn on the MRI for selected structures. A PSM was used to derive receptor density and apparent affinity from the [^18^F]-FMZ PET data.

**Key Findings:**

The PSM was found to adequately model [^18^F]-FMZ binding *in vivo*. There was a significant decrease in hippocampal receptor density in the SE group (p<0.01), accompanied by an increase in apparent affinity (p<0.05) compared to controls. No change in cortical receptor binding was observed. Hippocampal volume reduction and cell loss was only seen in a subset of animals. Histological assessment of hippocampal cell loss was significantly correlated with hippocampal volume measured by MRI (p<0.05), but did not correlate with [^18^F]-FMZ binding.

**Significance:**

Alterations to hippocampal GABA_A_/cBZR density and affinity in the post-kainic acid SE model of TLE are detectable *in vivo* with [^18^F]-FMZ PET and a PSM. These changes are independent from hippocampal cell and volume loss. [^18^F]-FMZ PET is useful for investigating the role that changes GABA_A_/cBZR density and binding affinity play in the pathogenesis of TLE.

## Introduction

Temporal lobe epilepsy (TLE) is the most common form of partial epilepsy in adults, and is often resistant to pharmacological therapies. A common neuropathology observed in patients with TLE is sclerosis of the hippocampus and the surrounding mesial temporal lobe. A decrease in inhibitory GABAergic neurotransmission in the hippocampus has been proposed to play a key role in epileptogenesis in TLE [1]. This reduction in GABAergic neurotransmission may be caused by any or all of the following: loss of GABAergic interneurons, loss of GABA_A_ receptors, or changes to GABA_A_ receptor subunits, leading to alterations in receptor properties [2], [3], [4] and needs to be further investigated. Previous animal and human studies have shown decreased GABA_A_/central benzodiazepine receptor (GABA_A_/cBZR) density in structures important to seizure generation in the mesial temporal lobe [5], [6], [7], [8], [9].

Radiolabelling of the GABA_A_/cBZR antagonist flumazenil (FMZ) has long been used to image GABA_A_/cBZR. Initially this was [^3^H]-FMZ for *in vitro* and *ex vivo* autoradiography assays, and subsequently radiolabelled FMZ conjugates were developed for *in vivo* clinical studies with PET. The most commonly used GABA_A_/cBZR-specific PET radioligand has been [^11^C]-FMZ [6], [7], [9], [10], and PET studies using this radiotracer in patients with chronic TLE have consistently shown reduced GABA_A_/cBZR binding in the hippocampus ipsilateral to the seizure focus, when compared with the contralateral side [10], [11], [12]. Further, studies that have coregistered [^11^C]-FMZ PET images with volumetric MRI have shown that decreases in GABA_A_/cBZRs are greater in magnitude than the decreases in hippocampal volume [13], indicating that the decreased receptor density is not merely a reflection of cell loss [10].

Despite the promising findings of both animal and patient studies, [^11^C]-FMZ PET has not been adopted for routine preclinical or clinical use due to a number of practical limitations. Firstly, [^11^C] has a short radioactive half-life, thus requiring an onsite cyclotron and dose by dose production. To overcome this there has been a recent focus on developing [^18^F]-radiolabelled FMZ conjugates, including [^18^F]-FEFMZ, [^18^F]-FFMZ and most recently [^18^F]-FMZ [14], [15], [16]. [^18^F] has a substantially longer half-life, meaning it does not need to be produced on site, and can therefore be used in a greater number of centres and is more practical for routine investigational use.

A second issue preventing the routine preclinical or clinical use of FMZ PET is that the standard modelling protocols require highly invasive serial arterial blood sampling for plasma input function into compartmental models, which is not ideal for longitudinal preclinical studies or a routine clinical diagnostic tool [17]. Insertion of arterial lines for arterial blood sampling requires local anaesthetic due to pain associated with placement of the cannula, further, the presence of the cannula can lead to infections, haematoma and bleeding following removal of the cannula. Modelling methods have been developed to analyse receptor-based PET data without arterial blood sampling, such as the simplified reference tissue model [18] but only give quantification of the binding potential (BP) which is the ratio of B_max_/K_d_. Previous work has shown that [^11^C]-FMZ PET quantification using the partial saturation model allows identification of both B_max_ and K_d_ [19]. Briefly, this method relies on the observation that flumazenil kinetics in cerebral tissue achieve a dynamic “Scatchard-like equilibrium” after the injection of a mass that ensures at least 50–70% occupancy of the receptors. This method is also non-invasive when a reference region is used to estimate the concentration of radioligand in the free compartment. Given that it has been shown that [^18^F]-FMZ kinetics are comparable to [^11^C]-FMZ in the human [33] we want to use the partial saturation approach (PSA) with [^18^F]-FMZ in rats. The PSA would allow the derivation of the concentration of GABA_A_/cBZRs (B_max_), and the apparent binding affinity of FMZ for the GABA_A_/cBZR (1/K_d_V_r_ with K_d_ being the dissociation constant and V_r_ the volume of reaction) in a single experimental protocol.

Before using the PSA, it is necessary to first re-validate the method for use with [^18^F]-FMZ, using the olive nucleus as a reference region. To do this, we perform a full identification using a multi-injection protocol with an arterially sampled input function, on which we can base simulations of a partial saturation experiment to test the robustness of the data analysis method. The multi-injection protocol was first reported in baboons using [^11^C]-FMZ and four injections [20]. The multi-injection protocol has not been explored in the rodent for [^11^C]-FMZ, however, multiple injection studies in the rat have been published using other radiotracers [21], [22], and there have been a small number of studies using a multi-injection protocol in baboons [23], [24].

The aim of this study was to investigate whether [^18^F]-FMZ PET, analysed utilising the partial saturation model, could be used to quantify binding parameters (receptor density and apparent affinity) for the GABA_A_/cBZR *in vivo* in the well validated post-kainic acid (KA) status epilepticus (SE) rat model of TLE. The first step was to validate the use of the partial saturation model for quantifying [^18^F]-FMZ binding parameters *in vivo* using a multi-injection protocol with arterial blood sampling. Finally, it was investigated whether *in vivo* assessment of GABA_A_/cBZR was correlating with neurodegeneration in the epileptic animals.

## Methods

### Animals

Nineteen (4+15) male in-bred Wistar rats were used in this study. The animals were single housed in standard opaque plastic cages with food and water *ad libitum*. They were maintained on a 12 h light/dark cycle (lights on at 6.30 a.m.) at 22°C with 60% relative humidity. The animals were treated in accordance with the Australian NH&MRC Code of conduct for use of animals in research and the study protocol was approved by the University of Melbourne Animal Ethics Committee.

### Tracer preparation

[^18^F]-FMZ was prepared according to Dedeurwaerdere et al [15]. Radiochemical purity ranged from 95–99% and specific activity ranged from 62.9–177.6 GBq/µmol at end of synthesis.

### Validation of the partial saturation approach using the Multi-Injection protocol

#### Animal preparation

Healthy rats (n = 4, 12–14 weeks, 333±24.7 g) were studied for the validation of the partial saturation model, using a multi-injection protocol and arterial blood sampling. Rats were anaesthetized with ketamine (100 mg/kg) and xylazine (20 mg/kg) injected intraperitoneally (i.p). The right femoral artery was cannulated using PVC tubing (Microtube extrusions Australia, id 0.4 mm, od 0.8 mm, 30 cm length). Briefly, a skin incision of about 1.5 to 2 cm was made on the right inner thigh. Blunt dissection was used to reveal the femoral artery and femoral vein located between the muscles. Approximately 1 to 1.5 cm of the artery was then separated from the vein and cleared of surrounding connective tissue with blunt dissection. The artery was then tied off both proximally and distally using silk suture (3.0 silk, Dysilk, Dynek Pty Ltd, Australia). Subsequently, a small incision was made on the wall of the artery under a dissecting microscope. The cannula, prefilled with heparinised saline (20 units) was tunnelled through the artery towards the heart (about 3–4 cm). Good placement of the arterial cannula was confirmed if blood was seen pulsating back and forth in the line, after which the distal sutures were tightened to secure the cannula in place. The rat was subsequently moved onto the imaging bed in a supine position to easily allow access to the catheter for arterial blood sampling and dorsal penile vein for [^18^F]-FMZ administration.

#### PET image acquisition and analysis

Data sets were acquired on a dedicated saPET scanner (Philips Mosaic). Under ketamine/xylazine anaesthesia, animals were PET scanned during a three-injection protocol (Table 1): i) a tracer dose of [^18^F]-FMZ (∼0.6 nmol, 59.9–69.6 MBq in 0.1–0.3 ml of [^18^F]-FMZ at t = 0 min), ii) co-injection of [^18^F]-FMZ and non-radiolabelled FMZ (4.0–4.1 nmol, 53.7–69.9 MBq in 0.15–3 ml, t = 42 min; and iii) a displacement injection of non-radiolabelled FMZ (330 nmol, 0.2 ml, t = 70 min).

**Table 1:** Injected FMZ at each phase of the multi-injection protocol.

Rat	Injection 1 (Tracer [^18^F]-FMZ)	Injection 2 (Co-injection: [^18^F]-FMZ and FMZ)	Injection 3 (Displacement: FMZ)
**A**	59.94 MBq	53.65 MBq	0 MBq
	0.6 nmol	4.0 nmol	330 nmol
**B**	65.49 MBq	57.72 MBq	0 MBq
	0.6 nmol	4.0 nmol	330 nmol
**C**	60.68 MBq	69.93 MBq	0 MBq
	0.6 nmol	4.1 nmol	330 nmol
**D**	69.56 MBq	66.71 MBq	0 MBq
	0.6 nmol	4.1 nmol	330 nmol
**Mean**	**63.92 MBq**	**62.00 MBq**	**0 MBq**
	**0.6 nmol**	**4.05 nmol**	**330 nmol**

The scan acquisition consisted of three successive phases per tracer injection: i) 6 min of list mode acquisition (starting 45 s before tracer dose injection) followed by dynamic mode acquisition (3×3 min); ii) 7 min of list mode acquisition (starting 105 s before co-injection) followed by dynamic mode acquisition (3×3 min) and iii) 7 min of list mode acquisition (starting 105 s before displacement) followed by dynamic mode acquisition (3×3 min).

During the scanning protocol, the foot-pad reflex was checked regularly and additional anaesthesia administered if necessary (25 mg/kg ketamine and 5 mg/kg xylazine, i.p). Heart rate and blood oxygen levels were continuously monitored using a pulseoximeter (Smiths Medical Pm Inc, Wisconsin, USA).

PET dynamic series were reconstructed with an ordered subset estimate maximization (OSEM) algorithm (four iterations and eight subsets). No corrections for attenuation or random events were applied. Each frame of the dynamic series was corrected for radioactive decay and calibrated in Bq/ml.

#### Image analysis

PET scans were analysed using Analyze 7.0 (Mayo Clinic, AnalyzeDirect, Inc, KS) and co-registered to a MRI template (T2-weighted acquisition, matrix: 294×286×277, voxel size: 0.23×0.23×0.23 mm) with intra-operator reproducibility of 0.5–1 mm. On the MRI-template, whole brain (1739 mm^3^), left (51 mm^3^) and right (48 mm^3^) hippocampi, and olive nucleus (8 mm^3^) were delineated as regions of interest (ROIs, [15]). The mean activity/pixel (0.23×0.23×0.23 mm) in each ROI was computed for each frame, resulting in the production of time-activity curves normalised to the injected activity and converted to pmol/ml.

#### Arterial blood sampling

Arterial blood samples (50 µl, 21 per phase or 63 samples in total) were taken every 15 s for the first 4 min and at 6 min, 10 min and 16 min during the scan after every injection (three times). The cannula was flushed with 0.2 ml of heparinised saline at the end of continuous sampling (4 min) and following the 6, 10 and 16 min blood samples for each injection, and animals injected with 1 ml of saline (s.c) following cessation of blood sampling to replenish lost fluids. The blood samples were collected into pre-weighed eppendorfs containing 50 µl of ice cold NaF (2.1%) to prevent further metabolism of the FMZ in the blood by esterases [25]. At the end of the experiment samples were stored on ice until weighing, followed by further storage at −80°C until the time of analysis.

Within 5–45 min of withdrawal, the whole blood samples were counted for radioactivity in a cross calibrated gamma counter.

Data were processed by subtracting the mean background activity (counts per minute, CPM) from the measured activity in the sample and the counts were decay corrected to the start of the respective injection time.

#### Data analysis

The complete identification of the parameters was done for each time activity curve for the hippocampus and olive nucleus extracted as described above. The estimation was done using modelling software developed in house with a commercial software package (Matlab 6.1, The MathWorks, Inc., Natick, MA, 2000) using a two tissue compartment (free ligand in the tissue, and specifically bound ligand in the tissue), five parameter model with the arterially sampled input function to fully identify all parameters (B_max_, K_1_, k_2_, k_on_/V_r_, k_off_).

#### Simulation of the partial saturation experiment

Based on the complete identification of the parameters, we were able to validate the modelling approach of the partial saturation model. The simulated kinetic curves for the hippocampus (target region) and the olive nucleus (reference region) were generated. The simulation model used the arterially sampled input function, a two tissue compartmental (2TC) model for the target region and a one tissue compartmental (1TC) model for the reference region (free ligand in the tissue only, no specifically bound ligand). The simulation studies allowed us to validate that, using a mass of 4.5 nmol of [^18^F]-FMZ, a Scatchard like-equilibrium state occurs during the scan.

#### Partial saturation approach

B_max_ and K_d_Vr was estimated for each ROI using a nonlinear fit of the bound ligand (B) versus the free ligand (F) concentration: B = (F*B)/(F+K_d_Vr). For the validation of the PSA, the parameters were estimated using the true F from the simulated hippocampus as well as testing the method with the simulated reference region as the F.

#### HPLC – MS/MS analysis

Collected rat blood samples (40 µl) were diluted with water (80 µl), 1.0 M carbonate buffer (100 µl, pH 8.0) and 10 ng/ml zolpidem (50 µl, internal standard, IS). The mixture was subjected to liquid-liquid extraction with 50% ethyl acetate: 50% diethyl ether (3 ml). The organic layer was transferred, concentrated to dryness and reconstituted in mobile phase (100 µl). Chromatography was performed on X Bridge C18 column 2.1×30 mm, 3.5 µm (Waters Corp., Rydalmere, NSW, Australia) at a temperature of 30°C. The mobile phase consisted of 0.1% formic acid in 2 mmol/l ammonium acetate (solvent A) and 2 mmol/l ammonium acetate and 0.1% formic acid in methanol (solvent B). A step gradient at a flow rate of 0.4 ml/min was used with a resultant analysis time of 6.0 min. Mass spectrometric detection was by selected reaction monitoring in positive-electrospray ionisation mode (FMZ *m/z*304.1→258.2; IS *m/z*308.2→235.0). The method was linear from 0.25 to 500 µg/l (r^2^>0.990). Inter-day accuracy and imprecision, over the analytical range, was 91.6 to 105.2% and <12.2%, respectively (n = 5). The signal-to-noise at the lower limit of quantification was approximately 12∶1.

Data processing: a correction factor (FMZ conc/[Actual blood vol (ml)/Actual blood volume (ml)+ NaF vol(0.05 ml)]*[supposed blood vol (0.05 ml)/supposed blood vol (0.05 ml)+NaF (0.05 ml)) was applied to the mass spectrometry data (ng/ml) to take into account the exact amount of the blood in the sample.

### Epilepsy study

#### Induction of status-epilepticus

Fifteen male in-breed Wistar rats (10 weeks, 268±4 g) were used in this study. SE was induced using a repeated low-dose KA treatment protocol, adapted from that of Hellier et al. [26], as previously described by our group [8], [27], which is associated with reduced animal mortality as compared to traditional single dose protocols. For this, eight male Wistar rats were injected intraperitoneally (i.p.) with an initial 5 mg/kg dose of KA (Ocean Produce International, Shelburne, NS, Canada) and then observed behaviourally by an experienced investigator for the presence of SE (loss of consciousness was confirmed by lack of response to repeated application of external stimuli). If sustained SE did not occur within 30 minutes, further 2.5 mg/kg doses were administered at 30 minute intervals until this occurred. The median dose of KA administered was 13.75 mg/kg, range 7.5–25 mg/kg. The severity of SE was similar between the rats despite the variability of doses of KA administered; consistent with previous reports noting that the dose of KA required to induce SE varies considerably between animals [28]. Control rats (n = 7) received saline injections only. Four hours after the induction of SE (or saline injections) animals were given a single dose of diazepam (2.5 mg/kg, i.p) to terminate SE.

#### MRI scan

Five weeks post-SE, 2–5 days before PET scanning, a volumetric MRI scan was acquired for volumetric analysis of brain regions and co-registration of the PET data. T2 weighted MR images were acquired using a 4.7 T Bruker Biospec 47/30 Avance small animal MRI scanner (Ettlingen, Germany), running Paravision 3.0 data acquisition (Ettlingen, Germany). Animals were anaesthetised using isofluorane (induction dose 5%, 1.5% maintenance dose, 1∶1 air/oxygen). After scout images were obtained, a T2-weighted image of the rat brain was acquired using a fast spin-echo sequence (repetition time (TR) = 3.1 ms, echo time (TE) = 67.5 ms; 256×256×27 matrix, 0.23×0.23×1 mm).

#### PET scan

[^18^F]-FMZ radiochemistry was performed as previously described [15]. Radiochemical purity ranged from 95–99% and specific activity ranged from 62.9–177.6 GBq/µmol at end of synthesis.

Six weeks following induction of SE, animals were placed in supine position in a saPET camera under ketamine/xylazine anaesthesia (75 mg/kg and 10 mg/kg, i.p., respectively) [15]. Simultaneously, the animal was given a single bolus injection of [^18^F]-FMZ (3.6–4.6 nmol, 43.66–77.33 MBq, 0.2–0.4 ml) via the dorsal penile vein as the scan commenced. The dynamic scan was acquired as 2×30 s, 2×1 min, and 10×3 min frames (total scan duration was approximately 45 min).

#### Image analysis

**Figure 1:**
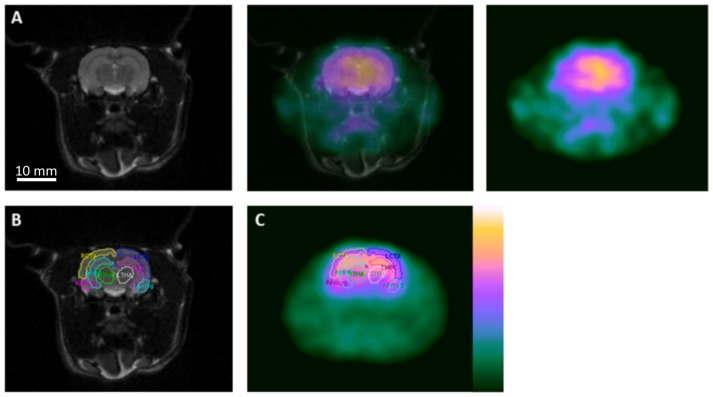
Coregistration and region of interest delineations for image analysis. (A) Coregistration of [^18^F]-FMZ PET with T2 weighted MRI, (B) Delineation of regions of interest on MRI (RCTX right cortex, LCTX left cortex, RHPC right hippocampus, LHPC left hippocampus, RTHA right thalamus, LTHA left thalamus, RAmyg right amygdala, LAmyg left amygdala), (C) Application of regions of interest on to [^18^F]-FMZ PET image.

PET dynamic series were reconstructed as described above. Analyze 7.0 (Mayo Clinic, AnalyzeDirect, Inc. KS) was used for analysis and manual co-registration (intra-operator reproducibility of 0.5–1 mm) of MRI and PET scans. On the MRI, the hippocampus, cortex, amygdala, thalamus, olive nucleus and ventricles were delineated as regions of interest (Figure 1). The mean activity/pixel (0.23×0.23×1 mm) in each ROI was computed for each frame, resulting in the production of time-activity curves converted to pmol/ml.

#### Data analysis

The concentration of free radiotracer in the tissue was approximated by the activity measured in the olive nucleus, a structure relatively devoid of GABA_A_/cBZR [29]. This structure has been selected because its remote position ensured a low level of spillover from other very specific regions, such as the hippocampus and cortical areas. B_max_ and K_d_V_r_ was estimated for each ROI using a nonlinear fit of the bound ligand (B) versus the free ligand (F) concentration: B = (F*B)/(F+K_d_V_r_).

#### Video-EEG monitoring

Within three days of the PET scan, each animal was implanted under ketamine/xylazine anaesthesia (as described previously [9]) with four epidural EEG screw electrodes (bilaterally in the frontal, parietal regions), and reference and ground electrodes (occipital region). For postoperative analgesia, an intraperitoneal injection of carprofen (4 mg/kg, Rimadyl, Pfizer, USA) was given and 1 ml of subcutaneous saline (0.9% NaCl) to prevent dehydration.

**Figure 2:**
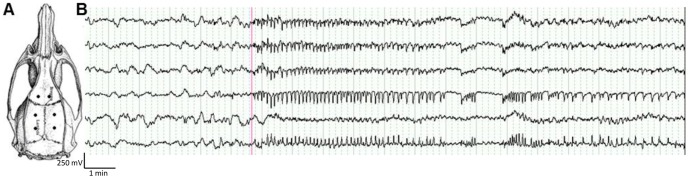
A typical seizure on EEG in a post-KA SE rat. (A) Position of recording electrodes on rat skull (adapted from Paxinos and Watson [46]). (B) The EEG trace shows a typical seizure in a rat 6 weeks post-KA SE demonstrating the abrupt onset of evolving rhythmic spike and wave activity from normal drowsy background EEG. Recording voltage is 50 µV/min.

One week following surgery, animals underwent five days of continuous video-EEG monitoring to confirm the occurrence of spontaneous recurrent seizures. Video-EEG recordings were made using the Compumedics™ system (Compumedics, USA) running Profusion EEG (version 3.7) with video recordings made using a Pentax digital colour CCD camera. EEG recordings were manually analysed using Profusion EEG. A seizure was defined as a paroxysmal electrographic discharge lasting longer than 5 s in duration which showing a clear rhythmical pattern distinct and at least 3 times the amplitude of the background rhythms (Figure 2). Seizures identified on the EEG recordings were then observed on the synchronised video and the Racine scale was used to classify seizure severity [30]. All post-kainic acid SE animals were observed to have at least one convulsive seizure during the five days of continuous monitoring (median 2, range 1–20 over 5 days).

#### Histology

Following the video-EEG recordings the animals were euthanized using a terminal dose of anaesthetic (Lethabarb 3.25 mg/kg, Virbac Animal Health, Australia). Brains were extracted and snap-frozen in isopentane cooled by liquid nitrogen, then stored at −80°C until sectioning for thionin staining.

Thionin staining was performed as previously described and two blinded investigators (TOB and SD) visually scored neuronal cell loss in CA1, CA3, CA3c and the dentate hilus [22], [31]. Cell loss was assessed on 6–8 sections of the hippocampus, −3.80 mm from bregma. This scoring scale ranges from 0–4 with 4: no neuronal loss and 0: complete cell loss.

#### Statistical analysis

Unpaired t-tests were used to assess significant differences between the control and KA-treated groups. Spearman's rank correlation coefficient was used to assess correlations between data.

A value of p<0.05 was considered significant. Values 0.05<p<0.10 were reported as trends to significance. PET data are expressed as mean ± standard error of the mean (SEM) and MRI and cell loss data expressed median ± inter-quartile range to visualise the range within the results.

## Results

### Validation of the partial saturation approach using the multi-injection protocol

#### Multi-injection protocol

**Figure 3:**
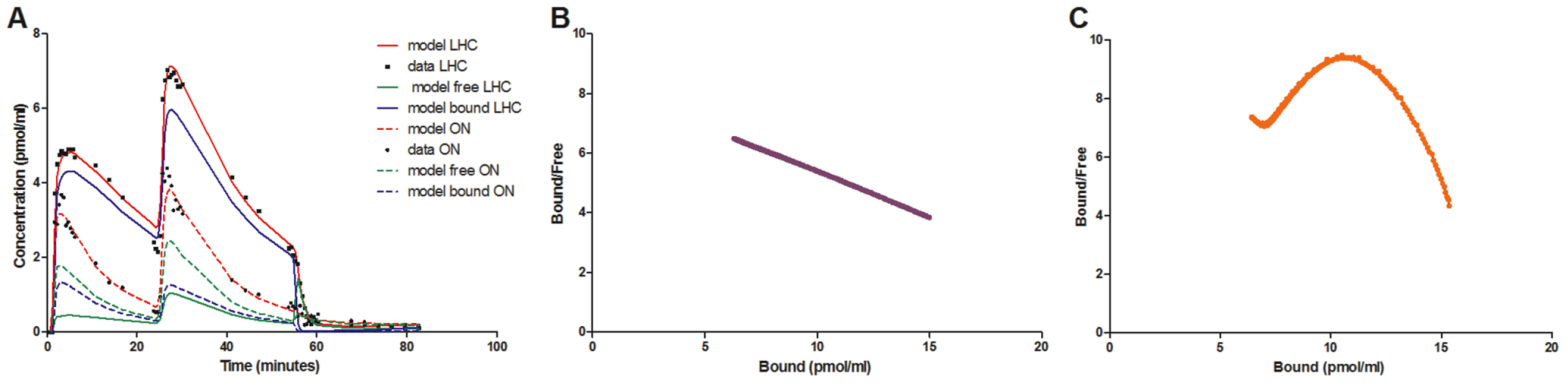
Kinetic curves from the multi-injection study and subsequent data simulations. (A) Individual data points for the left hippocampus (black squares) and the olive nucleus (black circles) are plotted, with the solid lines showing the model fit in the left hippocampus, and dashed lines the model fit in the olive nucleus. Red lines show total [^18^F]-FMZ uptake as estimated by the model, blue lines the amount of specific [^18^F]-FMZ binding, and the green lines the amount of free [^18^F]-FMZ in the tissue. (B) Scatchard plot for parameter estimates of the hippocampus using the true free fraction for the hippocampus. (C) Scatchard plot for parameter estimates of the hippocampus using the reference region (olive nucleus).

Figure 3A shows the time-activity curve for the multi-injection experiments in a representative animal. Total uptake in the hippocampi and olive nucleus were measured, and modelling simulations performed to fit the data to a two tissue-compartmental model. The results show an overestimation of the peak uptake in both regions, and a slight underestimation of specific binding in the olive nucleus (with a small amount of specific binding measured, which was not considered in the model).

Table 2 shows the binding parameters in the left hippocampus derived from the model for the four animals. Equilibrium constants between animals were consistent. Mean B_max_ was 30.85±2.73 pmol/ml in the left hippocampus, and 29.61±1.41 pmol/ml in the right hippocampus, and mean 1/K_d_V_r_ 0.30±0.05 pmol/ml in the left hippocampus and 0.33±0.03 pmol/ml in the right hippocampus.

**Table 2:** [^18^F]-FMZ binding parameters in the left hippocampus as derived from the partial saturation model using a multi-injection protocol.

Rat	B_max_ (pmol/ml)	K_1_ (pmol/ml)	k_2_ (pmol/ml)	k_on_/V_r_ (pmol/ml)	k_off_ (pmol/ml)	K_d_V_r_ (pmol/ml)	1/K_d_V_r_ (pmol/ml)	DV1 (pmol/ml)	BP
**A**	27.26	0.23	0.77	1.24	4	3.23	0.31	0.29	8.44
**B**	29.29	0.30	0.71	1.67	4	2.39	0.42	0.42	12.23
**C**	38.92	0.39	0.70	1.14	4	3.49	0.29	0.56	11.13
**D**	27.91	0.12	0.33	0.76	4	5.26	0.19	0.37	5.31
**Mean**	**30.85**	**0.26**	**0.63**	**1.20**	**4**	**3.60**	**0.30**	**0.41**	**9.28**

#### Simulations

Simulations of the partial saturation analysis experiment were performed to validate the use of the data analysis method. Parameter estimations using the true free fraction from the simulated hippocampus produced B_max_ values of 27.3 pmol/ml and 1/K_d_V_r_ of 0.31 pmol/ml when using the time-window 4.3–60 min (Figure 3B). These estimates were within <1% of the input parameters and therefore provided a good reference region could be found, the method will produce accurate parameter estimates. Using the reference region generated by a one tissue compartmental (1TC) model with no binding, and a time window of 5.8–60 min, the B_max_ and K_d_V_r_ values generated were 27.4 pmol/ml and 0.34 pmol/ml respectively, which are within <1% and 8.7% of the simulation input parameters (Figure 3C) Thus bias in the K_d_V_r_ estimate comes from using a reference region, however, the simulated reference region kinetic may not fully represent the experimental reference region kinetic as it was generated using a 1TC model, which may be the source of bias observed.

### The epilepsy study

#### Hippocampal GABA_A_/cBZR density and apparent affinity as measured by [^18^F]-FMZ PET

**Figure 4:**
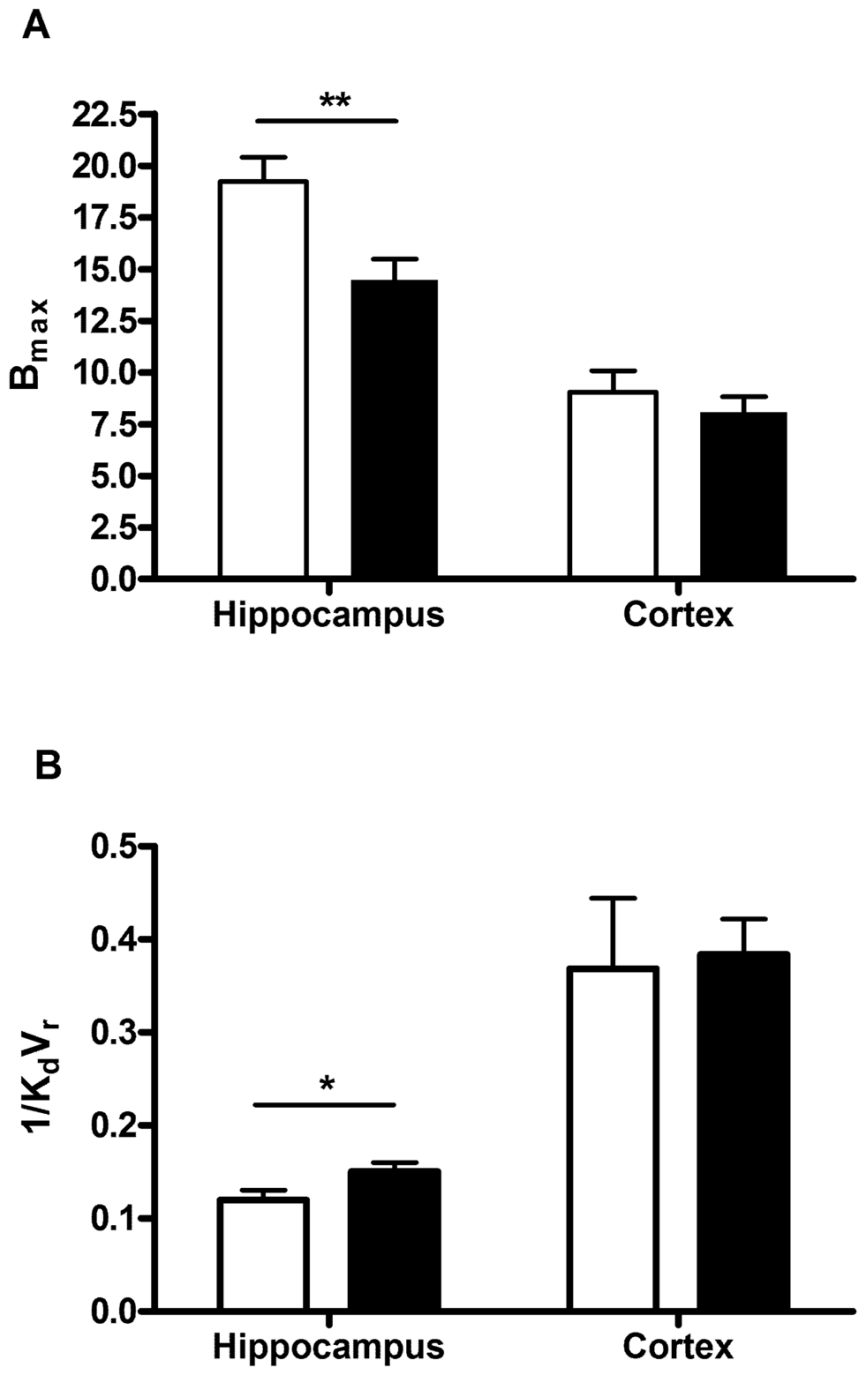
GABA_A_/cBZR density and apparent binding affinity in the hippocampus and cortex of epileptic and control animals as measured by [^18^F]-FMZ PET and the partial saturation model. Results are expressed as B_max_/1/K_d_V_r_ (mean±SEM, pmol/ml) in control (white bars, n = 7) and epileptic (black bars, n = 8) (**p<0.01; *p<0.05 epileptic vs. control); (A) B_max_ values 6 weeks post-SE (B) 1/K_d_V_r_ values 6 weeks post-SE.

Hippocampal B_max_ was significantly reduced in epileptic animals compared to controls (14.17±2.84 vs 19.23±1.18 pmol/ml, Figure 4A; p<0.01). Interestingly, the hippocampal B_max_ values in the control animals were lower than those observed in the healthy animals in the multi-injection study (30.85±2.73 vs 19.23±1.18 pmol/ml, p<0.01). A significant increase in apparent binding affinity (1/K_d_V_r_) was observed in the hippocampi of epileptic animals compared to controls (0.15±0.01 vs 0.12±0.01 pmol/ml, Figure 4B; p<0.05). No difference was observed in the GABA_A_/cBZR B_max_ or 1/K_d_V_r_ in the cortex between controls and epileptic animals (Figure 4A and B).

#### In vivo MRI volumetrics

**Figure 5:**
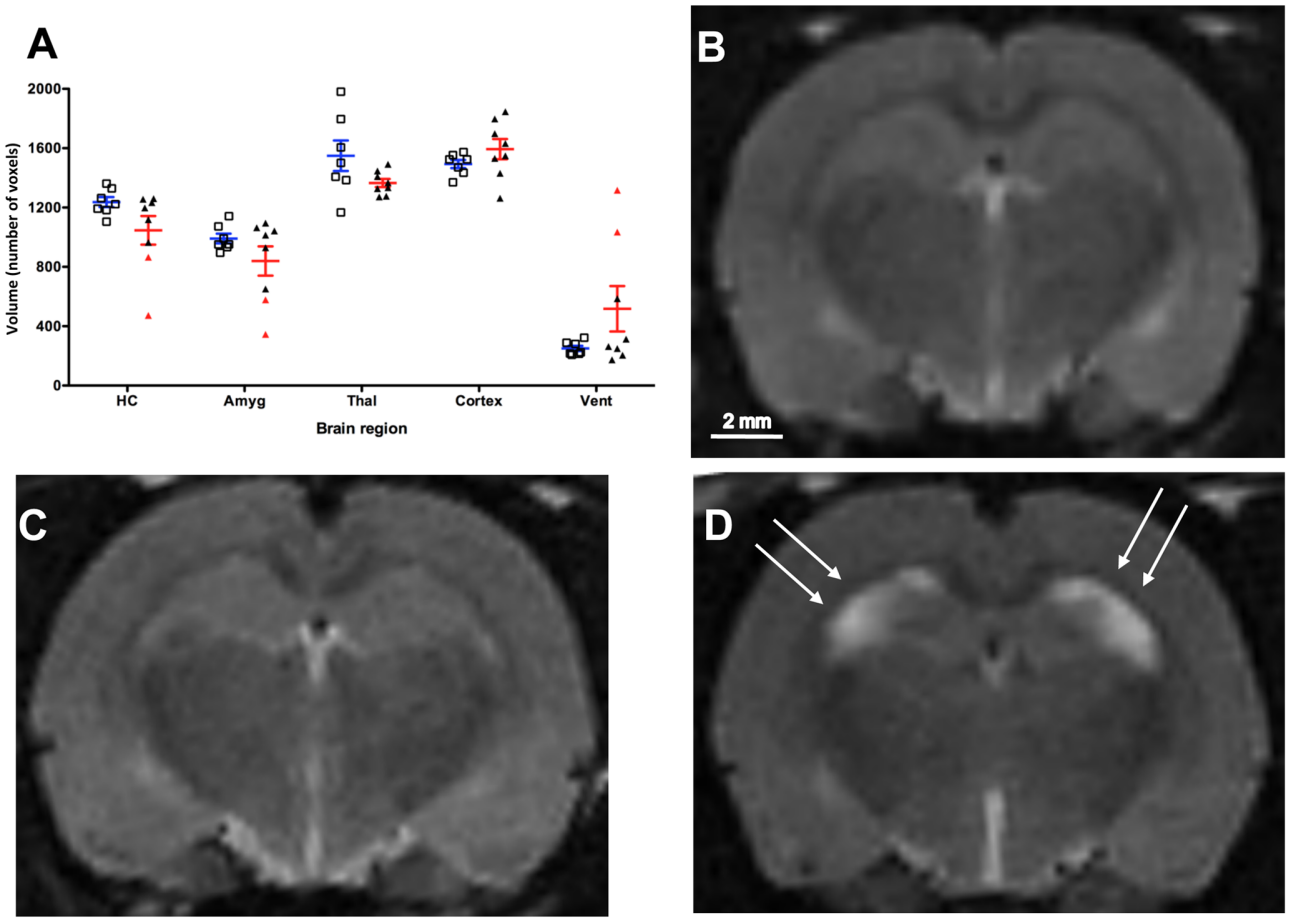
Volumes of regions of interest for epileptic and control animals as measured by manual delineation on MRI. (A) Results are expressed as volumes (number of voxels, median and inter-quartile range) in control (blue lines, n = 7) and epileptic animals (red lines, n = 8). Individual volumes are plotted for control (open squares) and epileptic animals (closed triangles). A subset of epileptic animals showed reduced hippocampal and amygdala volumes and increased ventricular volume (red triangles, n = 2) indicative of hippocampal sclerosis. Coronal slices through the hippocampi on MRI (B) a control animal, (C) an epileptic animal without hippocampal sclerosis, (D) an epileptic animal with hippocampal sclerosis. White arrows indicate decreased hippocampal volume.

Overall, there were no significant changes in MRI volumetrics for any brain region measured (Figure 5A). However, a subgroup of epileptic animals showed substantial limbic sclerosis, as shown by decreased hippocampal and amygdala volumes and increased ventricular volume (Figure 5A). Figure 5D shows an animal with reduced hippocampal volume, and increased ventricular volume, representative of limbic sclerosis.

#### Visual scoring of cell loss

**Figure 6:**
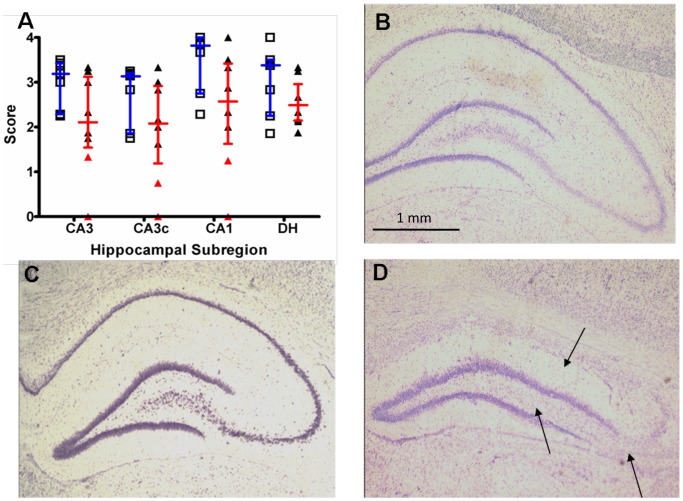
Cell loss as assessed by visual inspection in sub-regions of the left hippocampus 6 weeks post-SE. (A) Results are expressed as visual score of cell loss (median and inter-quartile range) in control (blue lines, n = 7) and epileptic animals (red lines, n = 8). Individual scores are plotted for control (open squares) and epileptic animals (closed triangles). A subset of epileptic animals showed considerably reduced scoring in CA1, CA3 and CA3c (red triangles, n = 2) indicative of hippocampal sclerosis. Thionin stained sections of the left hippocampus from (B) a control animal (C) an epileptic animal without hippocampal sclerosis/cell loss (D) an epileptic animal with significant hippocampal sclerosis/cell loss as indicated by the black arrows.

No significant differences were observed in visual scoring of cell loss in subregions of the hippocampus between epileptic and control animals (Figure 6A). As with the MRI data, there was a subset of epileptic animals who, compared to both control (Figure 6B) and non-lesional epileptic animals (Figure 6C), displayed severe cell loss similar to that seen in patients with hippocampal sclerosis (Figure 6D). However, when scores for individual regions were combined to give a total hippocampal cell loss score, there was a significant decrease in scoring in the epileptic animals compared to the controls (8.63 vs 13.83, p = 0.04).

#### Correlations analyses

**Figure 7:**
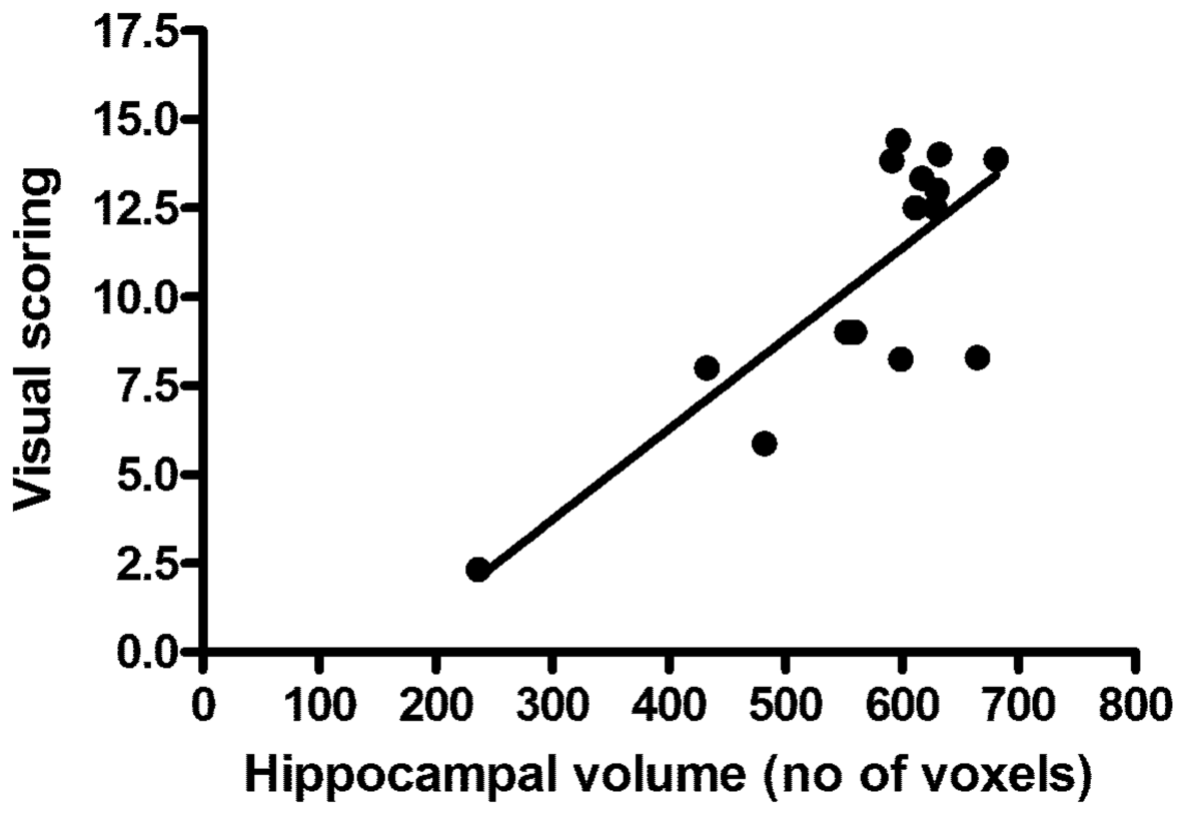
Relationship between whole hippocampal volume and cell loss in the left hippocampus. Spearman's correlations of whole hippocampal volume against summed visual scoring of cell loss in the left hippocampus. A significant correlation was observed (p<0.05, r^2^ = 0.60).

Hippocampal volume as measured by MRI showed a significant correlation with the summed score of hippocampal cell loss (r^2^ = 0.60, p = 0.02, Figure 7).

No correlations were found between cell loss or hippocampal volume and GABA_A_/cBZR density or apparent affinity (data not shown, all p>0.08, r^2^<0.22). Also seizure frequency did not show significant correlations with any structural or GABA_A_/cBZR measurements (data not shown, all p>0.15, r^2^<0.55).

## Discussion

This is the first study to utilise [^18^F]-FMZ PET to quantify GABA_A_/cBZR density and apparent binding affinity *in vivo* in an animal model of epilepsy. Previous animal studies utilising [^18^F]-FMZ PET have focussed on assessing tracer kinetics and metabolite profiles [15], [32]. More recent human studies have investigated modelling of binding potential (B_max_/K_d_) in healthy adults [33], [34]. The key findings of the study are as follows: (1) The multi-injection protocol allowed accurate modelling of [^18^F]-FMZ kinetics, validating the use of the partial saturation model; (2) A significant decrease in GABA_A_/cBZR density (B_max_) in the hippocampus was seen in epileptic animals compared with non-epileptic controls; (3) A significant increase in hippocampal GABA_A_/cBZR apparent binding affinity (1/K_d_V_r_) was observed in epileptic animals compared to controls; (4) No overall differences were observed in MRI volumetrics between epileptic and control animals; (5) Hippocampal volume correlated strongly with visual scores of hippocampal cell loss in the CA1, CA3 and CA3c; (6) The changes in GABA_A_/cBZR B_max_ and K_d_V_r_ in the hippocampus were independent of hippocampal cell and volume loss.

### Validation of the partial saturation model

The current study employed a multi-injection protocol with arterial blood sampling to model [^18^F]-FMZ kinetics using a standard two-tissue compartmental model in order to derive values for the validation of the non-invasive partial saturation model. This technique has previously been successfully employed to model [^11^C]-FMZ kinetics in humans [19], [35]. This validation allowed the use of the non-invasive partial saturation model in the epilepsy study. The benefit of this model over traditional models is that quantification of receptor density and affinity can be derived from a single-injection protocol without arterial blood sampling. This simplified protocol, used in combination with [^18^F]-FMZ, is appropriate for longitudinal studies. Most previous [^11^C]-FMZ PET studies have used compartmental modelling with arterial blood sampling [17]. Non-invasive models include the simplified reference tissue model [33], image derived plasma function compartmental modelling [36], Logan graphical analysis and multilinear reference tissue model [37]. However, these models derive either a volume of distribution (V_d_) or a binding potential (BP_ND_), not receptor density (B_max_) and affinity (1/K_d_V_r_). The partial saturation approach combines aspects of the simplified reference tissue model, with the partial saturation injection [35]. The method lends itself well to the performance of serial PET acquisitions to study the temporal evolution of the changes in GABA_A_/cBZR density and affinity *in vivo* following an epileptogenic brain insult.

### GABA_A_/cBZR density and affinity in an animal model of TLE

This study found a significant reduction in GABA_A_/cBZR B_max_ in the hippocampus of epileptic animals compared with controls as measured *in vivo* by [^18^F]-FMZ PET. This is in agreement with a number of previous [^11^C]-FMZ PET studies investigating GABA_A_/cBZRs in TLE patients. Only two studies in TLE patients have derived B_max_ from [^11^C]-FMZ PET. One study found reduced receptor density in the epileptogenic zone as seen on parametric images of B_max_ [38]. The second study also reported decreased receptor density (expressed as an asymmetry index), and associated this with a shorter inter-ictal period prior to the PET acquisition suggesting that this may be a transient phenomenon following seizure [10]. Another study, found binding potential was decreased in both the ipsilateral and contralateral temporal lobes of TLE patients when compared with controls [39]. Cortical GABA_A_/cBZR density did not differ between the control and epileptic animals indicating the reduction in GABA_A_/cBZR density is not a global brain change, which further supports a region specific pathophysiological down-regulation of GABA_A_/cBZRs in the hippocampus in response to epileptogenesis and/or seizures.

Despite the large number of studies looking at various [^11^C]-FMZ binding parameters, only one previous study has quantified binding affinity in an animal model of epilepsy, finding no difference in binding affinity (K_d_) between KA-treated and control animals despite reduced GABA_A_/cBZR density as measured by [^11^C]-FMZ PET [9]. An autoradiography study using [^3^H]-FMZ found decreases in both B_max_ and K_d_ in subregions of resected hippocampi following epilepsy surgery [5], again agreeing with the current study. In a previous study using *ex vivo* autoradiography in the post-KA SE rat model of TLE we found a decrease in the GABA_A_/cBZR density in hippocampal subfields but no change in receptor binding affinity (K_d_) in epileptic animals six weeks following the SE [8]. Consistent with this, in this *in vivo* study at the same time point post-SE we found a decrease in B_max_ for [^18^F]-FMZ binding however, in contrast to our *ex vivo* study and previous work by Syvanen and colleagues [9], we also found an increase in binding affinity (1/K_d_V_r_). This apparent disparate finding may be due to the measurement of slightly different parameters, with 1/K_d_ being a measure of affinity for the receptor, and 1/K_d_V_r_ affinity for the receptor as a function of the distribution of the radiotracer. Alternatively, changes in GABA_A_ subunit expression may explain the increase in affinity seen in this study. The central benzodiazepine binding site is found between the α1 and γ2 subunits. Despite both the α4 and α6 subunits being benzodiazepine insensitive, FMZ is known to be partial agonist of these subunits [40]. Studies using the post-kainic acid SE model of TLE have shown increases in both α1 and α4 subunit and mRNA expression, with decreases in α2 and α3 [41], [42]. Further, alterations in α subunit expression have been seen in the white matter of temporal lobe tissue following surgical resection [43]. Alterations in subunit composition have been shown to affect sensitivity to neurosteroids [44] and therefore subunit expression changes may alter [^18^F]-FMZ binding, reflected by the increased 1/K_d_V_r_ apparent affinity seen in this study.

In this study, MRI volumes and visual scoring of cell loss did not significantly correlate with GABA_A_/cBZR binding characteristics, suggesting hippocampal cell loss or atrophy is an unlikely primary cause for the decrease in GABA_A_/cBZR binding seen in the epileptic animals. Nevertheless, there were clear decreases in hippocampal volumes and cell loss in a subgroup of epileptic animals. This is a reflection of the clinical situation where hippocampal sclerosis is present in only a proportion of patients with TLE. It is noteworthy that the MRI hippocampal volumes strongly correlated with cell loss in the CA1, CA3 and CA3c subregions of the hippocampus, in agreement with previous studies showing visual scoring of cell loss correlated with stereological cell counting [45], however there was no relationship between B_max_ and either hippocampal volume or cell loss. This agrees with previous human data which found no correlation between GABA_A_/cBZR binding and hippocampal volume or cell loss in TLE patients [5], [13]. Moreover, previous studies have shown consistently reduced [^11^C]-FMZ uptake in TLE patients with normal MRI [10], [11], [39], supporting our finding of reduced receptor density in epileptic animals without hippocampal sclerosis. This indicates that the changes in GABA_A_/cBZR binding and affinity are independent of structural alterations.

### Methodological considerations

As shown in the results, the B_max_ values derived from the multi-injection study were higher than those in the control animals in the epilepsy study. One possible explanation for this disparity is the use of template vs individual MRIs for the coregistration. In the multi-injection study, a template MRI was used for the derivation of the regions of interest. This may result in a less accurate delineation of the olive nucleus, which may account for the overestimation of B_max_ values in this group compared with the control animals with individual MRI in the epilepsy study. Alternatively, it may be the B_max_ values in the epilepsy study are underestimated, due to the partial saturation model requiring estimation of the free fraction from the olive nucleus (which may be not completely devoid of receptors).

This study has shown that the partial saturation approach is an appropriate method for the derivation of receptor-radiotracer binding kinetics *in vivo*. There are a number of assumptions with this model. Firstly, that an equilibrium state is reached amongst tissues, which was true in the current study from 8–50 minutes. Secondly, free concentration in the target tissue can be estimated from uptake in a reference region which is devoid of specific binding – in this case the olive nucleus. The olive nucleus was shown to provide robust estimates of the free compartment in the current study – with a small amount of specific binding in the MI data, which can be attributed to spillover from surrounding regions due to the use of a template MRI (as discussed above). Following the verification of these assumptions, standard Scatchard analysis can be used to derive B_max_ and K_d_V_r_.

### Conclusion

In summary this study has investigated the application of [^18^F]-FMZ PET with the partial saturation model to study GABA_A_/cBZR density and affinity *in vivo* in rats. The results demonstrated a decrease in both GABA_A_/cBZR B_max_ and 1/K_d_V_r_ in the hippocampus of epileptic animals, in agreement with previous human and *ex vivo* animal studies of TLE. We have validated the use of [^18^F]-FMZ PET and the partial saturation approach for studying changes in GABA_A_/cBZR density and affinity *in vivo* and demonstrated the independence of these from structural changes in the hippocampus. This method does not require serial arterial blood sampling or multiple injections, making it ideal for longitudinal studies. Future studies could focus on longitudinal changes in GABA_A_/cBZR density and affinity during the epileptogenic process, providing new insights into the role of these changes in the pathophysiology of the disease, facilitating the development of [^18^F]-FMZ PET for clinical application.
